# Seasonal variations and risk assessment of microplastic contamination in agricultural soil and associated macroinvertebrates in Egypt

**DOI:** 10.1038/s41598-025-88715-9

**Published:** 2025-02-24

**Authors:** Safa M. El-masry, Azza M. Khedre, Asmaa N. Mustafa

**Affiliations:** Group of Invertebrates ecology and pollution - Department of Zoology, Faculty of Science, Sohag University, Sohag, 82524 Egypt

**Keywords:** Global problem, Microplastics, Terrestrial ecosystem, Soil fauna, Risk assessment, Pollution load, Ecology, Zoology, Ecology

## Abstract

Contamination by microplastics (MPs) has the potential to rank among the world’s most significant environmental issues. Despite the fact that MP contamination is a global problem, little is known about the time variation of MPs in agricultural soil and its faunal communities which represent a key role to risk assessment. This study represents a first field investigation regarding the MP concentrations in agricultural ecosystem in Egypt. Our study investigates the seasonal fluctuations of MPs in soil and its common fauna in a citrus orchard (*Citrus sinensis*) in Egypt’s Sohag Governorate. Moreover, this work aimed to identify how feeding strategies and body size of the selected fauna affect the no. of MPs ingested. The greatest mean concentration of MPs in soil was observed in summer (664 ± 90.20 items/kg) dry weight. However the lowest was recorded in autumn (354 ± 70.92 items/kg). *Aporrectodea caliginosa* (earthworms) was more contaminated with MPs (6.84 ± 2.5 item/individual annually) than *Anisolabis maritima* (earwigs) (2.06 ± 0.86 item/individual annually). When comparing between taxa without considering the size of the organisms, earwigs showed higher MPs concentrations (ranged from 117.93 ± 5.23 to 244.38 ± 4.57 items/gm wet weight) than the earthworms (ranged from 25.62 ± 2.43 to 51.66 ± 4.05 items/gm wet weight). Our results found that blue and red colors were the predominant colors in the soil and the selected fauna. Also, polyester fibers (PES) were the most popular type of microplastics, followed by fractions of polyethylene (PE) and polypropylene (PP). Interestingly, the reduction in the MP particles in the present taxa was observed compared to those in the soil. Pollution load index (PLI) value varied across seasons, with the lowest recorded in autumn due to reduced MPs abundance. The Hazard (H) index indicates a moderate risk (level III) due to high polyester abundance and a low hazard score (4) across all seasons. Our results represent a starting point for further studies on the impact of MPs on soil organisms in various agricultural soils.

## Introduction

Plastic materials have found applications in numerous strategic sectors including industry, agriculture, medicine, building, automotive manufacture and more, due to their low cost, flexibility, lightweight nature, stability and long-lasting durability^1–3^. The total annual production of plastics worldwide has reached approximately 350 million tons^4^. As a result, the widespread distribution and poor management of plastic waste has made it a global problem^3^. Plastic materials can remain longer in the environment because of their exceptional long-lasting durability and resistance to biodegradation. A variety of disintegration processes including weathering, photodegradation, mechanical abrasion, microbiological degradation, and others can fragment plastic wastes into microplastics (MPs; <5 mm)^5–7^.

Microplastics produced from primary and secondary sources, which produce plastic particles of varying sizes^8,9^. Primary MPs (artificial microplastics are generally derived from microbeads) are manufactured in extremely small sizes for specific uses including cosmetic products (creams, lotions and soap), toothpaste and related products^10^. Secondary MPs are very small pieces of plastics like fragments and films created when larger size of plastics are exposed to different degradation processes^10^. MPs can be categorized into a variety of shape, including fragments, fibers, films, foam, and beads^11^.

Up to date, the bulk of research on MP pollution has focused on the source, sink, fate, and biological effects of MP particles in aquatic ecosystems^12–14^. Microplastics can enter the terrestrial ecosystem via a variety of sources that are largely influenced by anthropogenic activities. These sources including irrigation water, agricultural production activities (the widespread use of agricultural plastic films, compost products and the organic fertilizers or sewage sludge), atmospheric deposition and automobile tire abrasion^3,15^. Recently, the soil contamination by MPs has gained increasing attention^6,16,17^.

Soil MP pollution has been determined as the second most significant scientific problem in the realm of environment and ecology^3^. According to researches, the overall amount of MPs in the terrestrial system (such as soils) may be 4–23 times higher than that in the ocean^3,6^. Annually, the amount of MPs in cultivated soil surpass that in the ocean, suggesting that soil serves as a bigger sink for plastic waste than the ocean^18^. Nevertheless, the monitoring data about the occurrence and distribution of MPs in soil settings are now severely deficient due to the lack of suitable analytical procedures to detect MPs in soils^2^. Elevated levels of MPs have been documented in several agricultural sites all over the world^3,19^. The presence of MPs in the agricultural soils has been shown to affect soil physical and chemical properties, soil functions, plant performance, biodiversity and microbial activities^2,3,20,21^.

Soil organisms might be accidentally ingested MP particles across the food chain^22,23^. Accumulation of MPs in the gut of organisms lead to potential harm effects such as growth decrease, reproductive reduction and even death^16,24^. These resultant effects are significantly correlated with the shape, size of MPs and type of polymer^23,25^. Although MPs have been detected in the bodies of some terrestrial species^26^, data remains limited regarding the relationship between the burden of MPs in soil fauna and environmental variables. Indeed, identifying ecological (habitat and trophic level) and biological (body size and feeding strategy) factors that influence the ingestion of MPs in soil fauna has a significant role in understanding their impact on soil taxa. This investigation may improve our understanding of the mechanism of piling up and transmission of MPs through the terrestrial food chain. Furthermore, our findings highlight the importance of investigating the ecological risk posed by MPs in polluted areas and raise awareness about MPs in agricultural ecosystems because agriculture is important for food security in Egypt.

In Egypt, no data is available on the concentration of MPs in agricultural soil or in faunal communities, especially under field conditions. This knowledge gap prevents a thorough understanding of the ecological and environmental risks associated with MPs. This study aimed to:

(i) investigate the seasonal concentration of MPs in soil and two different taxa of soil macroinvertebrates belonging to two classes (Insecta and Oligochaeta) in the Agricultural Research Center at Jazirat Shandaweel District, Sohag Governorate, Egypt.
(ii) detect the physical features (shape, color, size) and chemical structure of MPs.
(iii) investigate whether feeding strategies of the selected soil macroinvertebrates affect the number of ingested MPs.
(iv) determine whether organism body size affects MP load per individual among soil macroinvertebrates.
(v) evaluate the possible risk of MPs to agricultural soil.

## Materials and methods

### Site of Collection

**Fig. 1:**
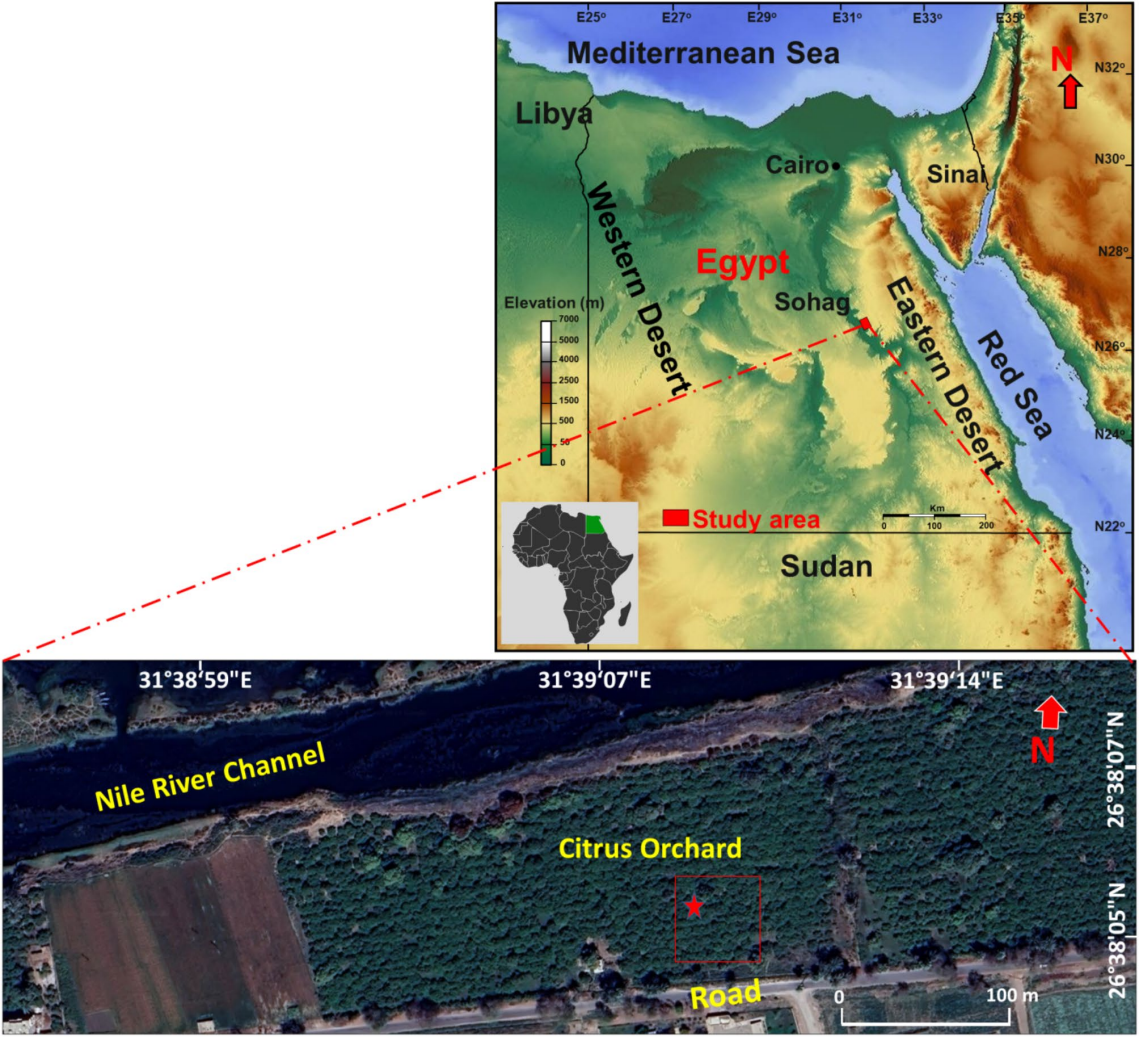
Egypt map showing Sohag Governorate with Google Earth photo showing collecting site (Star refers to the point of collection). Photographs showing the location of the sampling site within the Citrus Orchard. Images/Maps data: Google Earth and USGS Explorer.

**Fig. 2:**
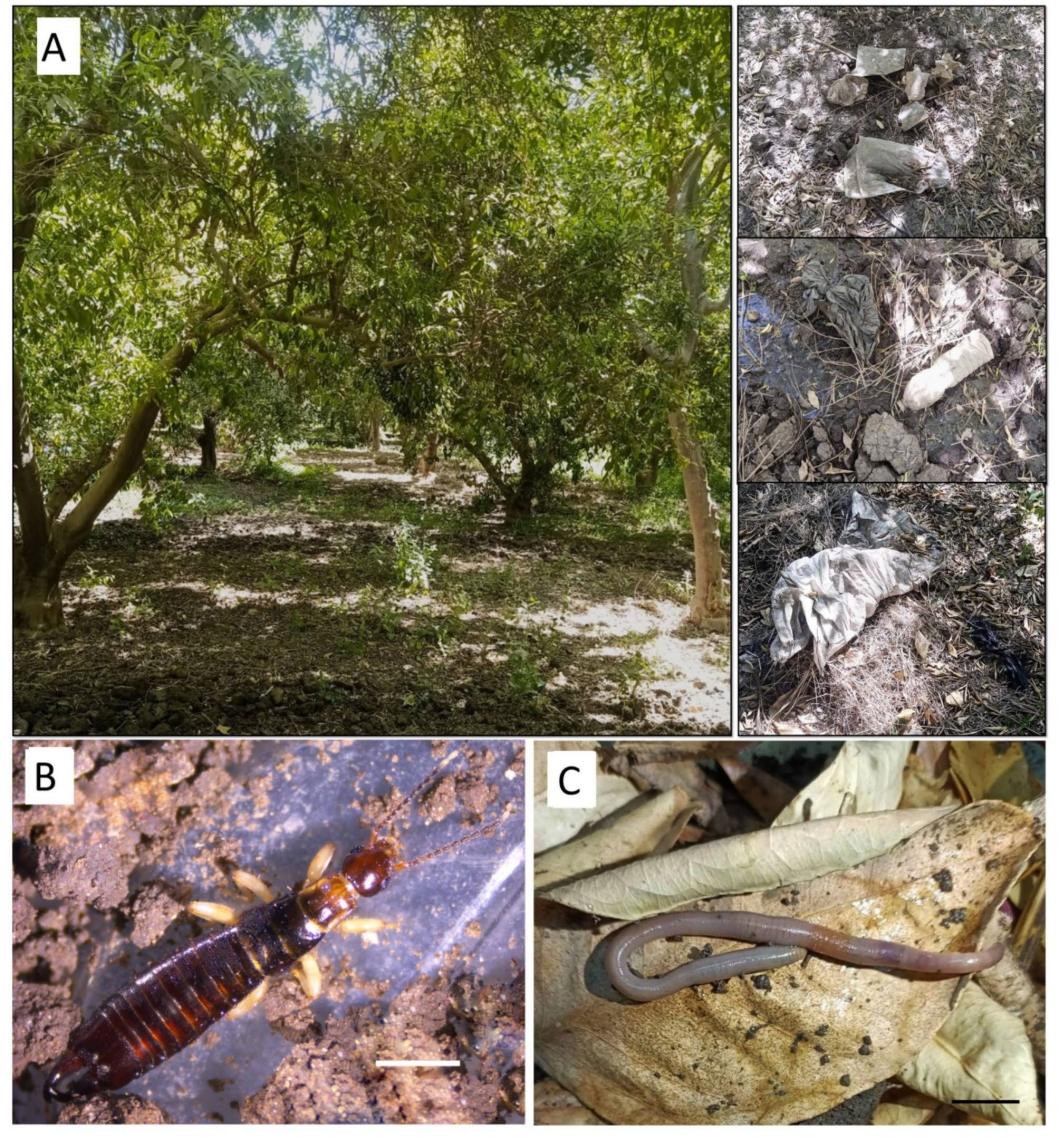
Photographs showing (**A**) types of pollution within the sampling point at the Citrus Orchard and soil fauna; (**B**) *Anisolabis maritima* (ASA/A/100); (**C**). *Aporrectodea caliginosa* (ASA/A/103) (Scale bar = 1 mm).

The research was conducted in a citrus orchard (*Citrus sinensis*) situated in the Agricultural Research Center at Jazirat Shandaweel District, Sohag Governorate, Egypt. The orchard is located approximately 12 kilometers to the north of Sohag city. It lies between latitudes 26º 37’41.65"N and 26º 38’ 7.59"N, and longitudes 31º 38’ 47.44"E and 31º 39’ 37.95"E. The study area covers a large portion of the Research Center. Within this space, a randomly selected part of this area measuring 50 × 50 m^2^ was chosen for examination. This study area is bounded by a road to the south and the Nile River channel to the north (Fig. 1). According to our previous published study^27^, the soil composition is classified as clay-loam soil (23.9% sand, 38.6% silt and 37.5% clay). The area is subjected to human activities such as agricultural practices (Fig. 2A).

## Sampling Plan and procedures

Over the course of a year, samples were gathered periodically from June 2022 to May 2023. The strategy of sampling in the present study aimed to collect representative samples across the agricultural field, thus five separate points in each season were chosen to require a comprehensive distribution pattern of MP contamination in the selected site. The collecting tool was an iron quadrate that measured 25 × 25 × 15 cm^27^. Each sampling point was obtained from the surface soil layer (0–15 cm depth) utilizing a stainless-steel spoon. Soil fauna were collected from the identical soil collection points, sorted and transferred to clean glass containers containing 70% alcohol to prevent the ejection of gut contents^28^. In the laboratory, the most abundant soil macroinvertebrates (*Aporrectodea caliginosa* and *Anisolabis maritima*) were chosen to conduct the present study (Fig. 2).

## Identification

The earwig (*Anisolabis maritima*) and the earthworm (*Aporrectodea caliginosa*) were identified using the keys and descriptions provided by^29–31^(Fig. 2B, C).

The identified specimens are lodged in the Zoology Department at Sohag University, Egypt. The form ASA/#/### was used for coding the specimens collected and they were deposited at Zoology Museum, Zoology Department, Faculty of Science, Sohag University, Egypt.

## Laboratory analysis

### MPs extraction from soil samples

The method of^32^ was applied with some modification to separate MPs from soil. The soil samples were laid in clean and covered glass bottles and thereafter dried for 48 h in an oven set to 60 °C. After drying, 50gm of each soil sample was placed in a clean 1 L beaker, and then MPs were separated from denser natural particles using a density separation technique. A freshly prepared hyper-saline solution of NaCl/NaI (density of 1.50 g cm^− 3^) was added to the soil samples until the contents of the beaker reached ~ 500 mL^33,34^. Before adding hyper-saline solution, 30 ml of 30% H_2_O_2_ was added to each beaker to break down any organic matter that may have been present. The beakers were placed on a shaker table (OS-2000, open-air-dual-action shaker, JEIOTECH, Korea) and shaken at 200 rpm for two days to separate any MPs hung in the soil samples. Then, the samples were left for 24 h to allow the MPs to float and the soil particles to precipitate. The floating supernatants were carefully poured into the funnel of the filtering pump, while the solid soil precipitates were washed to assure that all the remaining MPs were extracted. The supernatants were subsequently filtered through 0.45 μm filter paper to amass all the MP particles. After that, the original sample flask was filled again with a saturated NaCl/NaI solution, and the density separation and filtration procedure were carried out at least twice more, using a new filter each time. Finally, the MPs that precipitated on the filters were placed on clean and dry Petri dishes, which were covered and kept at room temperature in a desiccator. All the preceding processes were repeated several times to guarantee that all the MPs had been separated from the soil sample^35^.

### MPs extraction from soil fauna samples

According to^36^, depending on the size of each taxon, several individuals were grouped together to produce a sample for comparative analysis. For each season, ten samples, five from each selected taxa *(Aporrectodea caliginosa* and *Anisolabis maritima*) were prepared for determination of MPs. Each sample contained 3–5 individuals for *Aporrectodea caliginosa* and 5–10 individuals for *Anisolabis maritima*. All samples were cleaned carefully to remove external debris by deionized water. Then the individual of each sample were placed in filter paper to eliminate excess water. Before the digestion process, wet weight of each sample (gm) was measured. The individuals within each sample were placed in a glass beaker. To break down the biological tissues, various amounts of strong oxidizing acids were added for digestion, including 30% H_2_O_2_ (10–50 mL according to the size of individuals). Various physical techniques like heating and shaking were employed to optimize the effectiveness of purification. Heating typically took place in an oven at temperatures between 50 and 60 °C for a duration of 6 h. Each digested sample was filtered through filter paper (0.45 μm)^37^. Afterwards, filter papers were dried in an oven at 40 °C for 48 h and subsequently scanned for the presence of MPs.

Instead of assessing MPs based on the size of various soil fauna studied, researchers measured it per gram of wet weight of the organism. This approach enabled the comparison of different taxa while disregarding the organisms’ sizes^27,38,39^.

### Microplastic identification and characterization

All MPs were visually counted with the help Carl Zeiss dissecting microscope (X20) equipped with a digital camera. Shapes and colors of MP particles were identified and photographed as well. The Image J software (ver. 1.53f, see https://imagej.net/ij/) was used to measure the dimensions (diameter and length) of all MPs. An attenuated total reflection–Fourier transform infrared spectroscopy (ATR–FTIR, Alpha Bruker Platinum, 1-211-6353), using zinc slender crystal with an incident angle of 45 ± 15 and 560 scan time (24s) with a resolution of 4 cm^–1^, was used to validate the identification of MPs (range: 4000–400 cm^–1^). For the investigation, MP particles of various color and shape were chosen. The OPUS program was used to modify the data (Bruker Optics GmbH).

The polymer type was established via comparing the obtained spectra to the published reference spectra^40^. When the matching degree was > 80%, the result was considered credible.

### Microplastic risk assessment

#### Pollution load index (PLI)

 An integrated pollution load index (PLI) was calculated based on the degree of MP contamination in the agricultural soil samples^41,42^. PLI at each soil agricultural samples is related to MP concentration factors (CF_i_), as given below:

$$CF_{i} = C_{i} /C_{o}$$

$$\:\text{P}\text{L}\text{I}=\sqrt{\text{C}{\text{F}}_{\text{i}}}$$

Where C_i_ is the MPs concentration at each sampling point and C_o_ is the background MP concentration. Because of the absence of available background data, a reference background of 4.9 items/kg dry weight was adopted from^43–45^. PLI was divided into four classes to indicate the level of MP pollution by^14,42,45^: low (*<* 10), medium (10–20), high (20–30), and extremely high (*>* 30).

### Polymer risk assessment index

The polymer risk assessment index (H) was calculated according to^46^ using the following equation:

$$\:\text{H}=\sum\:_{\text{n}=1}^{\text{n}}{\text{P}}_{\text{n}}{\text{S}}_{\text{n}}$$

Where P_n_ is the ratio of each polymer type at each season and S_n_ is the polymer hazard score calculated by^47^, with PP = 4, PES = 4, and PE = 11.

^47,48^ divided H index into four levels: Level I: <10, Level II: 10–100, Level III: 100–1000, and Level IV: >1000.

### Potential ecological risk index

RI has been applied to assess the ecological and toxicological impacts of MPs^42,49,50^.

$$\text T_{\text i} = \text {H/C}_{\text i}.$$

$$\text {RI} = \text T_{\text i} \times \text {CF}_{\text i}$$

T_i_ denotes the toxicity coefficient of MPs. ^48,51^identified five contamination thresholds for RI which are as follows: level I is less than 150, level II is 150–300, level III is 300–600, level IV is 600–1200, and level V ismore than 1200.

### Precautions

Special care was given to prevent sample contamination, where cotton white lab coats and gloves were worn during the experiment. The extraction of MP was done in a sanitized environment, and all equipments used were regularly washed with distilled water. Samples were stored in a sealed glass Petri dish. In each season, 3 L of distilled water (filtered before use) was put in a glass bottle and sealed with a cap to avoid potential background contamination during laboratory analysis. The NaCl/NaI solution applied in the density separator was filtered before being used for soil and its associated fauna samples. To ensure accuracy, five sample blanks from each used solution in each season were also used as negative controls. The filter was examined using a stereomicroscope. As part of the visual observation process, three uncovered Petri dishes containing distilled water were positioned near the microscope as an airborne precaution.

### Statistical analysis

One-way ANOVA was employed to identify significant differences in microplastic-related data across groups, by using the IBM SPSS software (ver. 22, IBM Corp., Armonk, NY, USA). A statistically significant difference was accepted when *P* ≤ 0.05. Correlations between MP concentrations in soil and the selected biota were examined using a bivariate Pearson correlation coefficient.

## Results

### Seasonal abundance and characterization of MPs in soil

The investigation of blank samples showed very low background contamination of MPs (11 MPs in all investigated solutions). This level of contamination in blank samples was so few that their influence on the results is almost unnoticeable, thus no corrections were applied to the results. All the particles collected during the different seasons from the soil samples (504 particles) were analyzed by FTIR spectroscopy, where 98.22% of the analyzed particles were plastic polymers, the remaining 1.78% was cellulose-based materials and is not considered in the further presentation.

**Fig. 3:**
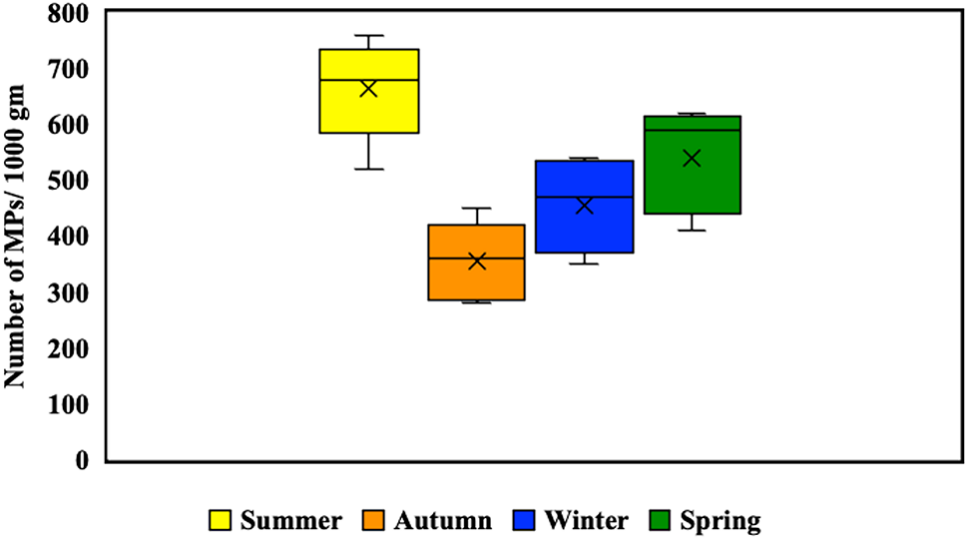
Mean seasonal abundance of MPs per kg of soil.

Regarding the seasonal distribution of MPs in the soil, the highest concentration of MPs was recorded in summer (664 ± 90.20 items/kg), while the lowest concentration was recorded in autumn (354 ± 70.92 items/kg) (Fig. 3). Statistically, the abundance values varied significantly from season to season (*P* < 0.05), with the abundance of MPs being significantly higher in summer compared to the other seasons (*P* < 0.05). Annually, the average of MPs abundance across the four seasons was 504 ± 140.50 items/kg.

**Fig. 4:**
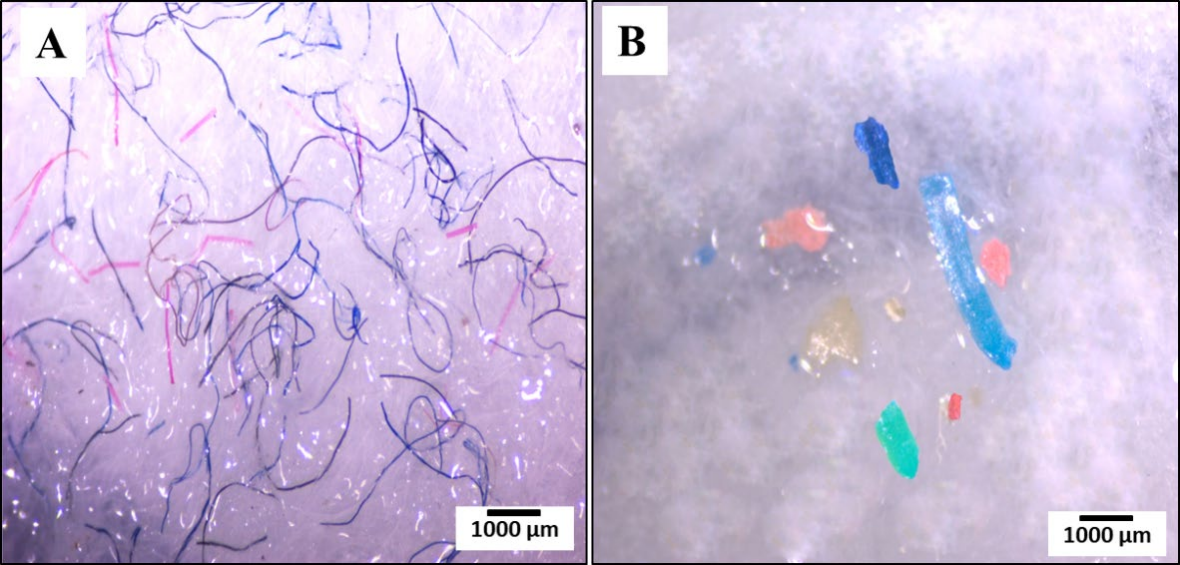
Photographs showing different shapes of microplastics obtained from soil and macroinvertebrates. (**A**) Fibers and (**B**) fragments. (scale bar = 1000 μm)

**Fig. 5:**
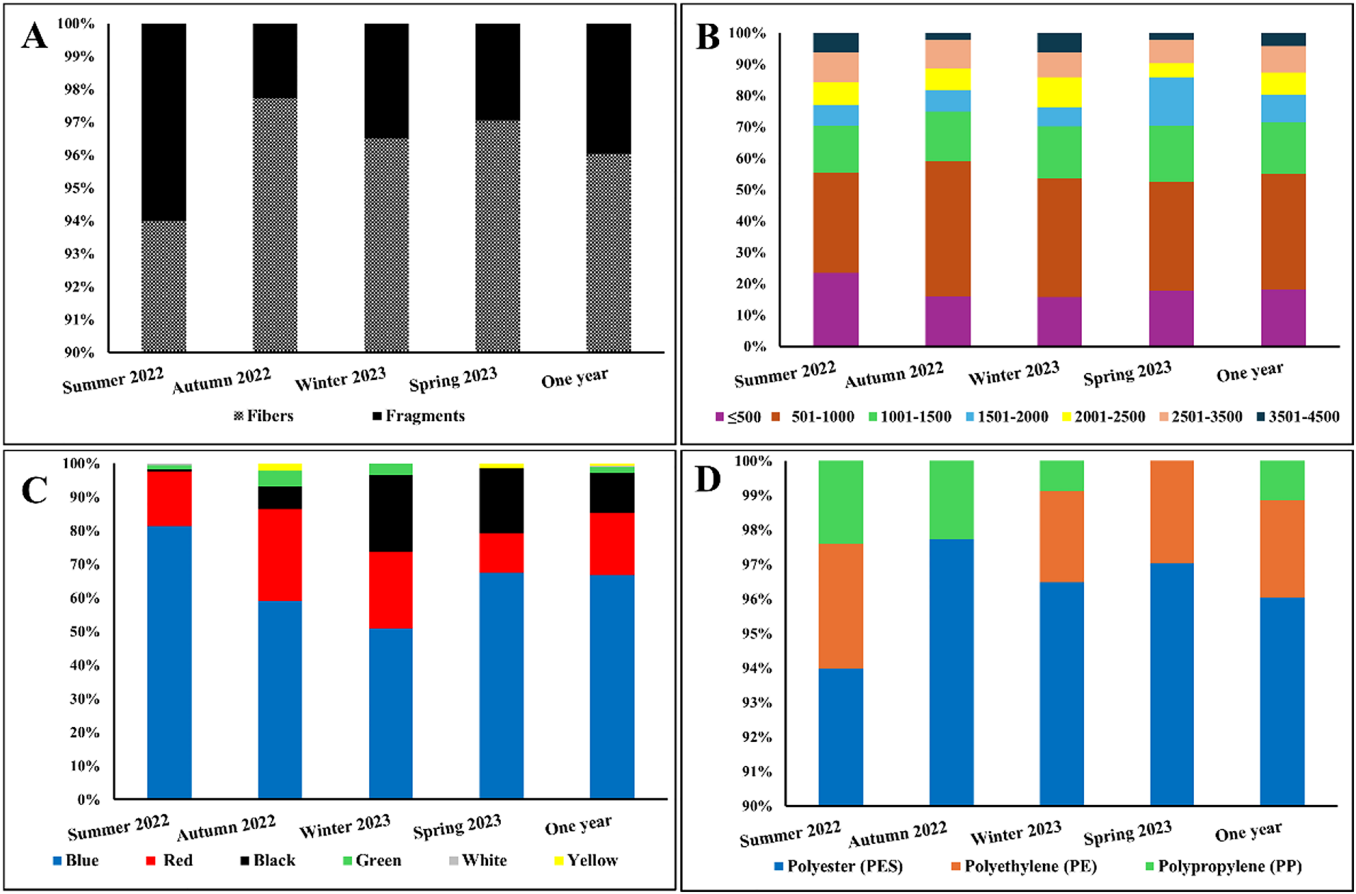
The percentage of the different microplastic shapes (**A**), lengths with µm, (**B**) colors (**C**), and chemical composition (**D**) collected from the soil.

Soil was contaminated with MPs and no MP-free samples were found among samples. The MP shapes detected in soil were fibers and fragments only (Fig. 4A, B). On an annual basis, fibers were the most commonly observed particles from soil samples, accounting for 96.03% of the total MP particles (Fig. 5A).

The soil MP particles length could be classified into seven size classes: ≤500, 501–1000, 1001–1500, 1501–2000, 2001–2500, 2501–3500 and 3501–4500 μm. The sizes of MP particles (fibers and fragments) collected from the soil did not exceed 4500 μm. The lengths of the fibers ranged from 152.03 to 4467.90 μm with an average of 1321.91 ± 966.68 μm, while the fragments ranged from 134.13 to 1041.84 μm with an average of 311.52 ± 168.76 μm. As for the width, the fibers ranged from 9.84 to 56.54 μm with an average of 19.89 ± 8.29 μm, and the fragments ranged from 110.70 to 603.13 μm with an average of 253.89 ± 116.79 μm. According to the MP size distribution across different seasons, the most abundant size classes of MPs were 501–1000 μm, ≤ 500 μm and 1001–1500 μm accounting for 36.91, 18.24 and 16.35%, respectively (Fig. 5B). Statistically, the yearly abundance of MPs in the range of 501–1000 μm was significantly greater than that of the other MPs size classes in the soil (*P* < 0.05).

Regarding to the MP colors, fibers and fragments exhibited a wide spectrum of colors. Six colors were detected including blue, red, black, green, white and yellow. Figure 5C shows the distribution patterns of MP colors in soil samples. The distribution of MP colors displayed seasonal variation in soil samples. The blue was the predominant color and accounted for the highest percentages (66.79%) among the samples detected in soil, followed by red (18.50%) and black colors (11.73%). The white color represented the lowest proportion among the other colors (0.20%). Statistical analysis showed that the annual abundance of blue MPs was significantly greater than that of the other MPs colors (*P* < 0.05).

**Fig. 6:**
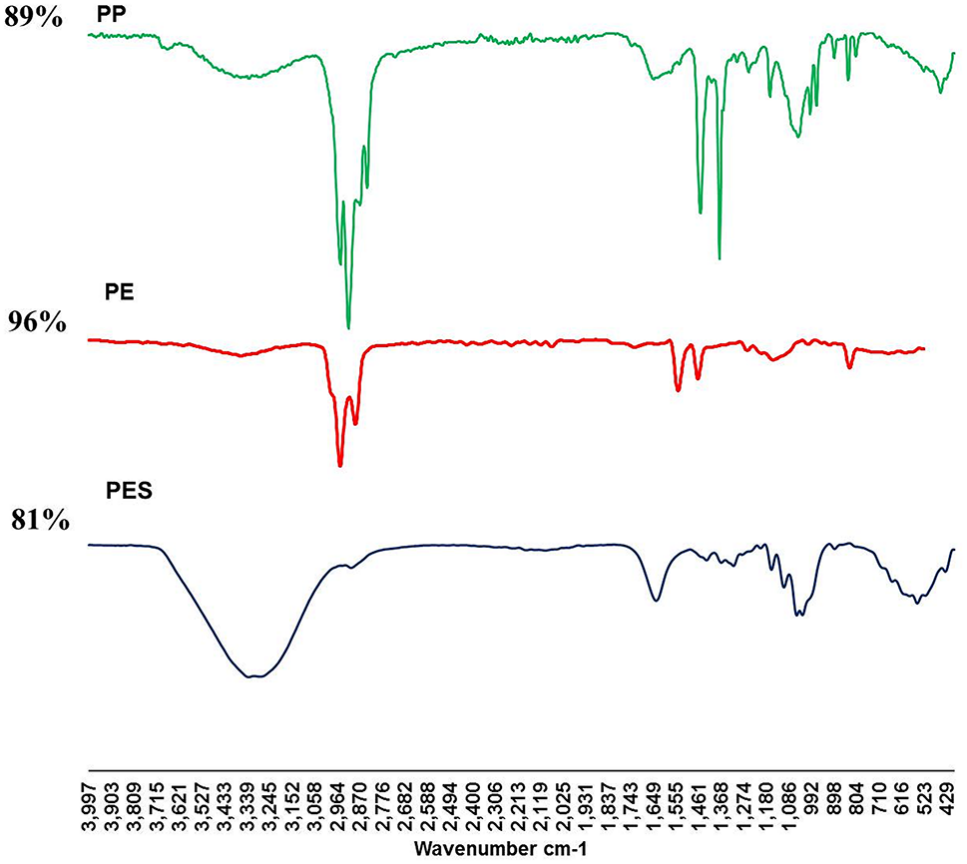
ATR-FTIR spectra of representative microplastic polymers extracted from soil and soil macroinvertebrates (PES, polyester; PE, polyethylene; and PP, polypropylene).

The MP particles collected from soil samples in this study were analyzed by FTIR spectroscopy to determine common polymers. The three polymer types were identified: PES, PE, and PP (Fig. 6).

Polymers of MPs collected from soil in the summer, autumn, winter and spring seasons were mostly PES (93.98%, 97.74%, 96.49%, and 97.04%, respectively). Statistically, higher significantly abundance of PES was found when compared with other polymers (*P* < 0.01). The distribution of polymer types in the different seasons was illustrated in Fig. 5D.

### MPs load indices

As illustrated in Table 1, calculated PLI values of the agricultural soil over various seasons were between medium to high, suggesting a moderate to high level of pollution discharge level (hazard category II and III). The maximum and minimum PLI values were also recorded in the summer and autumn, respectively. As a result, the obtained H values were nearly similar in the different seasons. A medium hazard to the environment is indicated by the H values of the polymers across various seasons, which fall under the category of III. Also, the MP RI index of the agricultural soil indicated a low degree of danger (degree I).

**Table 1:** Seasonal variations of MPs impact indices in the agricultural soil.

Seasons	CF_i_	PLI	H	T_i_	RI
Summer	135.51	11.64	425.32	0.64	664.00
Autumn	72.24	8.50	400.00	1.13	354.00
Winter	93.06	9.65	418.41	0.92	456.00
Spring	110.20	10.50	420.71	0.78	540.00
One year	102.76	10.14	419.80	0.83	503.50

CF_**i**_, Contamination factor; PLI, Pollution load index; H, Polymer risk assessment index; T_**i**_, Toxicity coefficient of MPs, and RI, Potential ecological risk index.

### Seasonal abundance and characterization of MPs in collected soil fauna

**Fig. 7:**
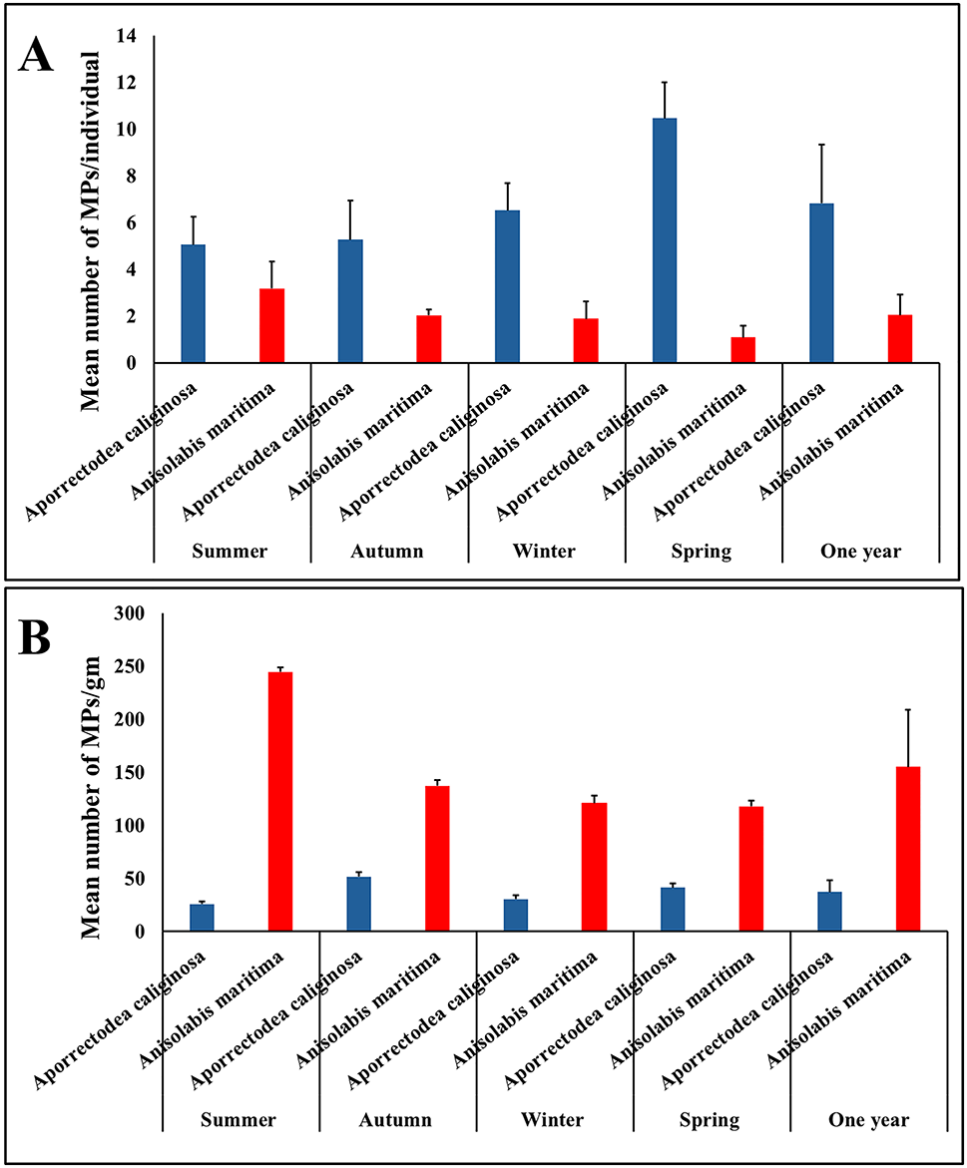
Mean seasonal abundance of MPs per individuals of soil fauna **(A)**, and Mean seasonal abundance of MPs per gm of soil fauna **(B)**.

The results reveled that there were no samples of macroinvertebrates fauna without MPs. During the present study, a total of 419 MP particles were collected from 62 individuals of *Aporrectodea caliginosa*. Regarding the average number of MPs per individual, the utmost values of MPs per individual were observed in spring (10.47 ± 1.54 items/ind), while the minimum value of MPs was observed in autumn and summer (5.28 ± 1.68 and 5.07 ± 1.19 items/ind, respectively) (Fig. 7A). Statistically, seasonal differences in MPs load per individual were significant (*P* < 0.05). Moreover, the MP load per individual was significantly greater in spring (*P* < 0.05). A total of 238 MP particles were obtained from 125 individuals of *Anisolabis maritima*. The maximum values of MPs per individual were observed in summer and autumn (3.18 ± 1.15 and 2.04 ± 0.26 items/ind, respectively), but the minimum value of MPs was observed in winter and spring (1.90 ± 0.74 and 1.10 ± 0.50 items/ind, respectively) (Fig. 7A). Significant differences in MPs load per individual was observed between summer and spring only (*P* < 0.05).

Because our measurements of body size for both taxa showed great variations between them, we calculated the number of MPs/gm wet weight to exclude the effect of body size on MP body burden. The result revealed that earwigs were more contaminated with MPs than those observed in earthworms in all seasons (Fig. 7B). Statistical analysis showed significant seasonal differences in MPs load/gm (*P* < 0.05) in both taxa. Moreover, the MP load per gram for *Aporrectodea caliginosa* and *Anisolabis maritima* was significantly higher in autumn and summer, respectively (*P* < 0.05). Annually, the average of MPs abundance was 155.07 ± 53.85 items/gm in *Anisolabis maritima* and 37.29 ± 10.8 items/gm in *Aporrectodea caliginosa* (Fig. 7B).

**Fig. 8:**
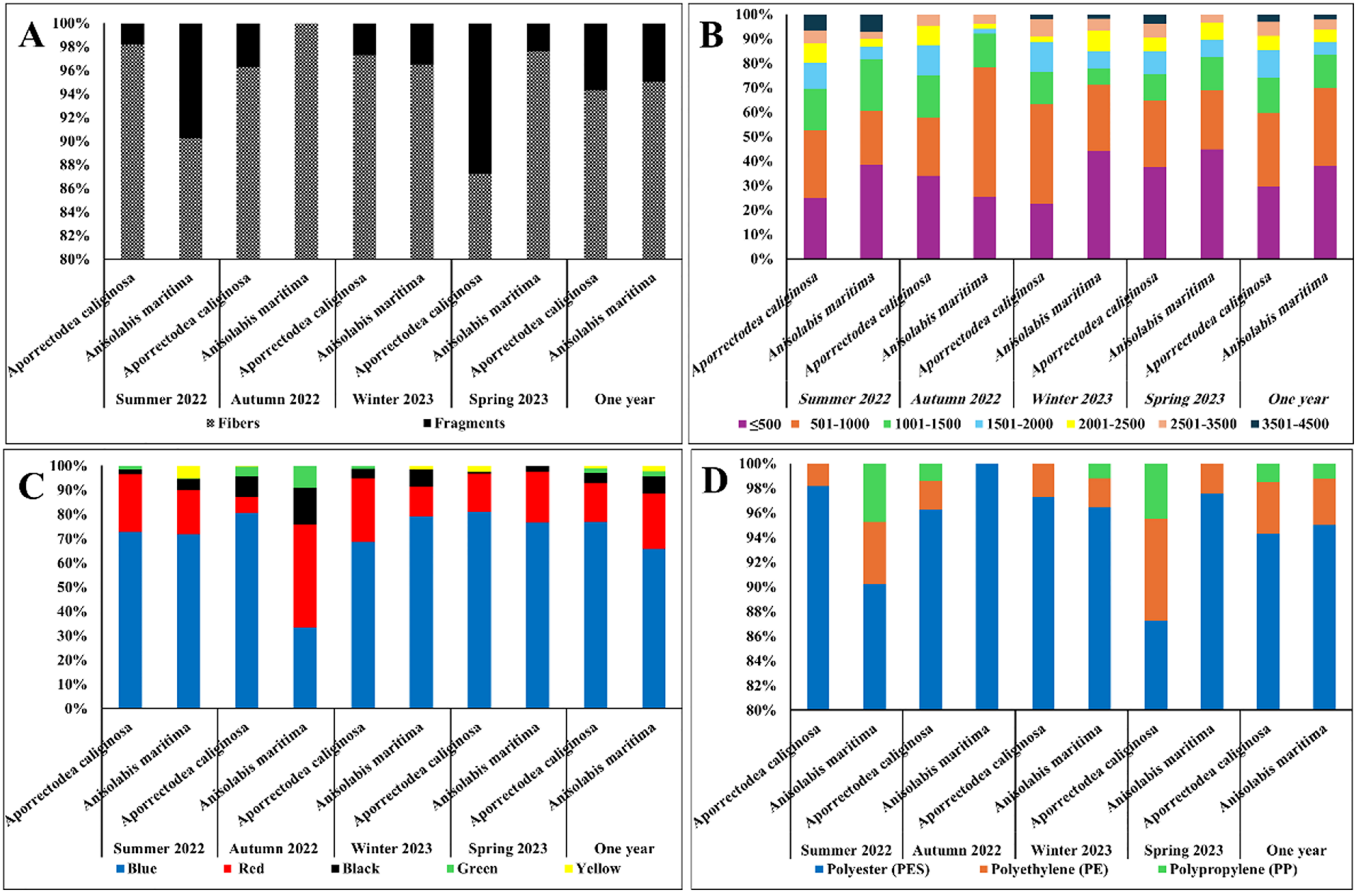
The percentage of the different microplastic shapes (**A**), lengths with µm, (**B**) colors (**C**), and chemical composition (**D**) collected from the selected soil fauna.

Most of the particles of MPs ingested by *Aporrectodea caliginosa* and *Anisolabis maritima* were fibers which represented 94.30% and 95%, respectively of the total ingested MP particles. While, the remaining proportions were fragments (Fig. 8A). Statistically, there are significant differences in the percentages of fibers and fragments were recorded in both species, throughout the whole monitoring year (*X*^*2*^ *= 0.000*,* P* < *0.05*). There are a positive significant correlation between the numbers of MPs per individual in *Aporrectodea caliginosa* and their size (*r* = 0.5, *P* < 0.05). However, this correlation was not significant with *Anisolabis maritima* where *r* = 0.37 and *P* > 0.05. Furthermore, there are no significant correlation between the numbers of MPs in *Aporrectodea caliginosa* and *Anisolabis maritima* and those found in soil (*r* = 0.08, *P* > 0.05, *r* = 0.2, *P* > 0.05, respectively).

Considering MPs length, the maximum length of the fibers collected from *Aporrectodea caliginosa* was 4257.73 μm and recorded during summer. The minimum length of these fibers was 107.31 μm and detected during autumn. While the lengths of fragments were ranged between 102.78 and 844.20 μm and recorded in spring and summer, respectively. While, the maximum length of the fibers in *Anisolabis maritima* was 4260.53 μm and recorded during summer. The minimum length was 108.56 μm and recorded during winter. While the minimum and maximum lengths of fragments were 98.40 and 314.77 μm, respectively and recorded in summer.

Considering width, the fibers collected from *Aporrectodea caliginosa* were ranged from 8.74 to 46.99 μm with an average of 20.11 ± 7.54 μm. While the width of fragments were ranged between 54.04 and 484.71 μm with an average of 145.53 ± 87.87 μm. Regarding fibers collected from *Anisolabis maritima*, they were ranged from 7.77 to 51.05 μm with an average of 19.02 ± 8.17 μm. While the width of fragments were ranged between 64.59 and 244.50 μm with an average of 133.44 ± 59.32 μm.

The percentages of the different MP size classes in the selected taxa showed fluctuations during the study period (Fig. 8B). Annually, the utmost abundant MP size classes in the earthworms were in the range of ≤ 500 and 501–1000 μm which were nearly similar percentages (29.78% and 29.93%, respectively). The MPs in the length range of 1001–1500 μm constituted 14.56% of the total. Statistical analysis showed that the annual abundance of MPs in the range of 501–1000 and ≤ 500 μm were significantly higher than those of the other MPs size classes in *Aporrectodea caliginosa* (*P* < 0.05). For *Anisolabis maritima*, annually MPs in the length range of ≤ 500 μm accounted for the highest percentage (38.19%), followed by the MPs in the length range of 501–1000 μm (31.60%) and 1001–1500 μm (13.88%) (Fig. 8B). Statistical analysis showed that the annual abundance of MPs in the range of ≤ 500 μm was significantly higher than that of the other MPs size classes (*P* < 0.05).

According to the MPs colors in *Aporrectodea caliginosa*, blue was the most observed color (76.84%), followed by red (16.01%), black (4.18%) and green (1.94%) (Fig. 8C). The yellow color represented the lowest proportion among the other colors (0.94%). Regarding *Anisolabis maritima* blue was also the most observed color (65.59%), followed by red (22.91%), black (7.03%) and yellow (2.33%) (Fig. 8C). The green color represented the lowest proportion among the other colors (2.14%). Statistical analysis for *Aporrectodea caliginosa* and *Anisolabis maritima* showed that the annual abundance of blue MPs was significantly higher than those of the other MPs colors (*P* < 0.05).

Polymers of MPs of *Aporrectodea caliginosa* in the summer, autumn, winter and spring seasons were mostly polyester (PES) (98.17%, 96.29%, 97.25% and 87.24%, respectively). PP was not detected in summer and winter (Fig. 8D). Also, PES has a highest abundance in of *Anisolabis maritima* in the summer, autumn, winter and spring seasons representing (90.23%, 100%, 96.48% and 97.58%, respectively). PP was not observed in spring and autumn, while PE lack in autumn (Fig. 8D).

## Discussion

### Concentration of MPs in the soil

Despite the absence of plastic mulching in our study location, we detected MPs in all collected soil samples. MP concentration ranged from 280 items/kg to 760 items/kg with a mean of 504 ± 140 items/kg throughout the period of investigation. This suggests that mulching films are not the only source of MPs in agricultural soils, and other MP sources influence MP soil concentrations. In Egypt, particularly in our study location in Sohag Governorate, the Nile River is the main source of irrigation water for most of the agricultural lands. The mean concentrations of MPs in the water and surface sediment samples of the Nile River in Sohag were reported to be 2.8 ± 0.93 items/L and 381 ± 113 items/kg, respectively^14^. This could directly affect the safety of irrigation water and explain the abundance of MPs in all soil samples. This is supported by^52,53^ who reported that the potential pathway for entrance of MPs into agricultural soil is irrigation, where contaminated water can participate in the release of MPs.

Another potential source for MP soil contamination is atmospheric deposition. Studies have shown that it can contribute a significant amount of MPs ranging from 136.5 to 152.0 MP/m^2^/day^54^. Both the proximity to urban areas^55^ and the distance from highways^56^ are likely to affect the extent of deposition. Considering that all the present soil samples were collected adjacent to the main road (< 50 m), there is a high possibility of MPs entering the soil through this source. Other MP sources are agricultural tools including irrigation pipes, fertilizer sacks, pesticide cans^57^, and plastic seed coating^58^. Moreover, plastic litter within the agricultural field and from the surrounding environment may be fragmented by environmental degradation, leading to the release of MPs to the soil^15^.

Furthermore, soil texture may be the most important factor influencing MP concentration. In our previous study^27^ ,we categorized the soil in our current study location as clay-loam soil according to the classification standard for soil particle grade. It contains a high clay fraction (37.5%) which makes it more stable and less susceptible to leaching. This likely results in a relatively high MP level in the surface layer of the soil. Additionally, the absence of heavy rains in Sohag Governorate may prevent the transport of MPs on the surface of the soil to other areas. In this regard, ^59^reported that MP concentration in soil varied depending on soil texture, where loose sandy soil contained fewer MPs compared to soils with finer fractions.

Reports have shown that MPs were detected in different types of agricultural soils from locations all over the world, such as Mexico, China and Bangladesh^60–63^. According to^64^results, MP concentrations in agricultural soils were in the range of 200–8000 items/kg. Moreover, the concentration of MPs has been reported in European agricultural soils and ranged from 1000 to 4000 items/kg)^62^.

Our results exhibited different MP concentrations compared to those recorded in different countries. Based on results from published studies, MP concentrations in land were high, and their distribution varied among different types of agricultural soil^65^ depending on anthropogenic activities^43^. The present data showed seasonal patterns in MP concentrations, as evidenced by the substantial variations observed within the sampled area. The highest mean MP concentration was recorded in summer (664 ± 90 items/kg), while the lowest was observed in autumn (354 ± 70 items/kg). This significant variability suggests that multiple factors likely contribute to the fluctuations in MP levels over the investigated period.

Additionally, the present study displayed that agricultural practices, such as irrigation and fertilizer application may introduce MPs into the soil at varying scales depending on the season, meaning that seasons in which the soil received more irrigation water and fertilizer may be exposed to higher numbers of MPs. In our study location, the field irrigation system was adjusted according to the season. Specifically, the soil was irrigated more heavily during the summer and spring compared to other times of the year. Moreover, fertilizer application was carried out primarily during the summer and spring seasons. ^66^suggested that the rate and frequency of fertilizer application can potentially affect the abundance of MPs in agricultural soils.

Another source that may contribute to the observed seasonal patterns in MP concentration is the increased traffic in roads during the summer. In this regard, ^43^reported that traffic activity may influence MP abundance in soil. Additionally, wind direction and speed, which impact atmospheric deposition may influence seasonal variation. This deposition is suggested to be responsible for the horizontal migration of MPs across geographical areas^67^. Seasonal variations in MP particles across agricultural soils have been recorded in previous studies^26,68^. ^26^reported that the MPs abundance in soil collected from fruit and vegetable farmlands in China was 56.67-180.33 items/kg for the spring samples and 206.15-890.49 items/kg for the winter samples.

The researchers suggested that these variations were principally associated with agricultural practices and meteorological circumstances throughout different seasons.

### Characteristics of MPs in agricultural soil samples

The most common shapes of MPs detected in agricultural soil samples globally were fragment, fiber and film^69,70^. Samples from previous studies often contained one, two or all three of the shapes. Similarly, the overall distribution of MP shapes in the present samples slightly varied among seasons mainly due to the increase in the numbers of fibers and decrease in fragments. The high percentage of fiber in the soil samples could be attributed to the prevalence of fibers in irrigation water. This may be supported by findings from a study that included samples from the Nile River where fibers accounted for 79% and 74% of the total MPs in water and sediment, respectively^14^. The authors reported that the increased presence of fibers in the Nile samples were related to certain human activities, specifically fishing processes and the discharge of domestic sewage into the Nile River. Increase in the proportion of fragments in summer compared to the rest of seasons indicates that human practices may also be an exporter for agricultural MP pollution^69^.

In all the soil samples, the most common MP particle size range was 501–1000 μm (≃37% of the total MPs annually). 35% of the soil samples contained MPs with a size range of 150–500 μm and 1001–1500 μm in 16.4% and 18.2% of the annual total, respectively. This may be referred to the fact that the plastic particles on the surface soil were exposed to more intense weathering (high temperature, UV radiation and mechanical abrasion) and thus aging^71^. Our findings were in accordance with most published studies that reported that the MPs collected from agricultural soils were predominantly ≤ 1 mm, most commonly, ≤ 0.05 mm^43,69^.

The chemical structure of the polymers refers to the various sources of plastic pollution^72^. PES was the main source of MPs and was widely distributed in our soil samples. Similarly, PES constituted the highest proportion of MPs obtained from Nile River water and sediment samples (79%; 74%, respectively) in a previous study^14^. This suggests that the irrigation water was the main source of PES in the soil samples. The higher concentration of PES in irrigation water could be attributed to its widely used in clothing, carpets and fishing nets^73,74^. The atmospheric fibers are a potentially important source of soil MP fibers^54^. While, PP and PE are the most applied plastics in urban areas and agriculture^75^.

In the present study, both PE and PP were present in lower proportions (2.83%, 1.14% annually), respectively. Different MP polymers have been detected in agricultural soils in previous studies including PP, PE, PES, PS and PVC^2,17,26^. The primary polymer found in the soils differed across locations indicating that the varying chemical composition is site-specific and dependent on agricultural practices more than population density as reported by^26^.

The color of MPs found in soil samples can provide a qualitative indication of their origin and age^76^. In this study, blue, red and black were the predominant colors in the soil samples. Our findings revealed that a considerable proportion of the MPs in the samples were colored plastics. Previous studies have identified various colors in soil samples, suggesting a diverse range of source for MPs^26,44^.

### Ecological risk

The ecological risk assessment of MPs required determination of the prevalence, characteristics, and types of polymers^77^. In our study, polymer type reflected the potential risk of MPs in agricultural soil samples. Our findings revealed a medium PLI over various seasons, and a lower PLI in autumn as it is associated with a lower concentration of MPs. Assessment of the ecological risk of MPs based solely on their concentration is unreasonable without evaluating MP chemical composition^78^ and the associated hazards of the detected polymers^47^. We found that, according to the PLI, the MPs detected in the agricultural soil had a moderate risk (level III) in all seasons. This may be returned to the high abundance of polyester, which has a low hazard score (4). Polymer chemical composition is a primary factor that impacts the hazard of MPs^79^.

However, soil had the lowest RI value (level I) indicating the low potential ecological risk of MPs distributed in the agricultural soil. In contrast, studies from other countries showed high MP pollution indices in agricultural soil^42,44^. Thus, calculating RI may help guide MP pollution control measures in agricultural soil.

#### MPs in terrestrial fauna

Regarding macroinvertebrate samples collected from the soil throughout the study period, we detected MPs in all samples despite their different taxonomic groups. This indicates that MP ingestion is not species-specific, and MPs may be detected in different taxa of terrestrial food webs. Our findings confirmed that MP body burden is highly variable and heterogeneous between macroinvertebrate orders as reported in other taxonomic groups^14,80^. The significant variations in MP body burden between the present taxa may be explained by differences in taxonomic position, size of the investigated organisms and interactions between them. Seasonal variation in MP body burden/individual in the present taxa may shed light on the contribution of different factors driving bioaccumulation including physiological conditions and metabolic rates which appear to impact the organism’s appetite and consequently their feeding behavior^81^.

Moreover, body size and longevity could be responsible for the apparent seasonal differences. Our findings revealed that there was a significant positive correlation between MP abundance/individual and the body size of earthworms. This may indicate that the size of this organism plays a crucial role in MP body burden. Several researchers have inspected the relationship between body size and the accumulation of MPs in organisms. For instance, studies have shown a positive correlation between biomass of freshwater invertebrates^82,83^ and fish^84^ and the number of MPs they ingested. The authors suggested that nutritional and physiological requirements of the organism were directly related to the amount of MPs they ingested. Moreover, a review of published results indicated that the size ratio between an organism’s body length and the highest number of MPs it can consume is approximately 20:1^61^.

Despite that the body size of earthworms influence the amount of MPs they ingested, we found no significant relationship between the size of individual earwigs and MP loads/individual. This result is in accordance with that of^85^ who reported that there was no relationship between the size (mass or length) of the individual Tubifex worms and MP body burden. More recently, ^86^found no correlation between the size of invertebrates collected from riverine ecosystems in Italy and the number of ingested MPs. They suggested that MPs passed through the guts of organisms from different taxa instead of accumulating in their bodies, resulting in the absence of an association between an organism’s weight and its MP load.

Because variable mean sizes were detected between the present taxa, we calculated the MP load relative to ww (gm) of both organisms to exclude the effect of organism size. Our results revealed that mean MP accumulation in earthworms was higher (6.8 ± 1.5 items) than that observed in earwigs (2.06 ± 0.8 items) when measured in terms of MP/individual. However, the number of MPs/body ww was higher in earwigs (155 ± 53.8 items/gm) compared to earthworms (37 ± 10.8 items/gm). This means that the ratio of MP load/gm is greater for smaller taxa. This is in agreement with^38^ who reported a reverse relation between MP concentration in organisms and their weight. Therefore, smaller organisms are more sensitive to MP exposure than larger organisms^87^.

It is evident that there are variations in MP accumulation between different taxa. These differences can vary depending on whether MP levels are measured in terms of number of items/ind or their concentration per body weight (MPs load/gm wet weight). Little is known about MP accumulation in soil taxa under field conditions. ^88^isolated 78 MPs from terrestrial snail samples, with a mean value of 1.77 ± 1.14 MPs/sample (each sample contained five snails) and an amount of 0.07 ± 0.01 MPs/gm. Moreover, ^26^found that the mean MP loads in earthworms in agricultural farmlands in China was 2.4 ± 0.1 items/gm.

In relation to the effect of seasonal variability on MP concentration, we found a significantly higher MP load/gm in earwigs (*A. maritima*) particularly in summer compared to the rest of the seasons. This indicates that temperature may affect the dynamics between prey and predator in the agricultural system^89^. Earwigs are similar to other predatory arthropods which consume their food indirectly through feeding on other organisms^90^. Therefore, higher MP load/gm in summer suggests that the consumption of the organism may increase with the increased temperatures. Increase in consumption could be attributed to a decrease in moisture content of both prey and predator at higher temperatures^91^. Previous studies have indicated that dermapteran insects required moisture for their development^92^. As a result, the insect may actively seek water supplementation by consuming more prey. It can be concluded that the relationship between predators and prey at various temperatures has a significant effect on MP load, particularly with respect to understanding predator-prey dynamics in terrestrial food webs.

Although in summer the highest MP concentration was detected in soil samples, the lowest MP load/gm body weight was observed in earthworms. Our finding confirmed the results of^93^ who reported that earthworms reduced their feeding activity below 10 °C and above 40 °C. This could explain why the higher MP load/gm was recorded in both autumn and spring where the temperature ranged between 15 °C and 30 °C.

According to our data, no significant relationship was observed between MP soil concentration and individual MP body burden in all taxa at different seasons. This indicates that MP ingestion and bioaccumulation by different taxa are not only affected by the presence of MPs in the terrestrial system, but also depend on combined biological factors associated with individual size^14,28^, life history traits^94^, feeding strategies^39,86^and egestion rates^28,95^. This suggests that environmental MP levels do not necessarily reflect the amount of MPs ingested by organisms in a specific area. Our results supported those of^96^ in which no significant relationship was found between the number of MPs ingested by aquatic invertebrates taxa and the average number of MPs in sediment or water of the corresponding sites. In contrast, a study by^97^ showed that MP body burden in mosquitofish (*Gambusia affinis*) was more correlated with the patterns observed in the water column^97^.

The soil fauna showed extensive distribution of fibers, which accounted for 78–100% of MPs, reflecting their high distribution in the agricultural soil. The shape of MPs ingested by the soil taxa was not taxon-specific, and the MPs were randomly ingested rather than selected from the environment. However, some freshwater invertebrates might selectively ingest microfibers instead of randomly uptake available MPs from the surrounding water^14^.

MP colors in the present taxa reflected their prevalence in the surrounding soil. Based on the results of^98^, earthworms have no obvious food choice. Thus, they might ingest MPs indiscriminately together with their food. Concerning predatory species (earwigs), the color of MP particles accumulated in their bodies more likely depended on the color of MPs retained in their preys as well as those ingested directly from the surrounding environment. This suggested limited feeding selectively for the color of most MPs in the predatory species^99^. Because both present taxa have limited feeding selectively based on feeding habits as previously mentioned, the distribution of MP size in their bodies followed more or less the same patterns observed in the soil. Therefore, our findings confirm the opinion that MP bioaccumulation is linked to their dominance in the habitat rather than organism preference^100^.

Interestingly, the percentage of the smallest MPs (≤ 500 μm) was significantly higher in both present taxa than those observed in the soil samples, representing 38% and 30% of the total MPs in earwigs and earthworms, respectively. It is important to emphasize that some particles of MPs detected in the earthworms and earwigs were smaller than those recorded in the soil samples. This finding supports the results of^101^ who observed a decrease in the MP particles in earthworm gut and suggested that this reduction occur through digestive activity which may be associated with worm gut microbiome including actinobacteria and firmicutes. Additionally, ^102^mentioned that the reduction in size of MP particles might be related to microbial enzymes in the soil that are ingested by the earthworms. Furthermore, ^103^reported that fractionating of MPs may take place mechanically through chitinous teeth in the gizzard Odonata nymphs. This hypothesis may be accepted to explain the noticeable reduction in the size of MPs particles found in the earwigs. In this regard, ^104^reported that the physical and chemical processes in food digestion can also affect the size of the consumed MPs, resulting in their breakdown into smaller sizes.

Detection of the chemical composition of MPs is very important to provide a better understanding of their negative effects on organisms^32^. The prevalence of polymer types in the present taxa reflects their abundance in the soil. PES was detecting in a higher proportion, representing 95% and 94% of the total MPs in earthworms and earwigs, respectively.

According to our findings from a previous study^27^, the soil in our study location is polluted with heavy metals (Cd, Pb, Cu and Zn). Moreover, according to the^105^classification, the ecological risk levels were moderate for Cd, Zn and Pb, and extremely high for Cu. MPs can absorb heavy metals from the soil^106,107^, and the concentrations of the heavy metals on the surface of MPs are higher than those in the surrounding habitat^108^. These two factors may have a synergistic effect on environmental pollution^26^. Additionally, heavy metals were detected in soil organisms in our study location^27^. This may result in adverse effects on organisms through bioaccumulation, which may cause harmful effects on the terrestrial ecosystem and human heath across the food chain^17,23^ .

## Conclusion

Despite MP pollution being a global issue, research on the temporal distribution of agricultural soil MPs remains insufficient. This is the first study to determine seasonal changes in relation to the concentration of MPs in soil and its organisms in an agricultural location in Egypt’s Sohag Governorate. MP contamination levels in the soil were higher in summer compared to other seasons. Furthermore, this study is the first to document the bioaccumulation of MPs in earwigs. MP abundance/individual was positively correlated with the size of soil organisms, and earthworms had higher MPs/individual than earwigs. However, MP body burden in the soil taxa was not related to the level of MP soil pollution because it is influenced by other factors, such as feeding strategy, organism size, and physiological conditions.

Blue polyester fibers were significantly more prevalent than other MPs in both soil and organism samples. Finally, our findings provide information about how seasonal variation can impact MP concentrations and their associated risk levels in terrestrial ecosystems. This may assist our government authorities to develop effective strategies for managing and mitigating plastic waste in the terrestrial environment.

Future monitoring is recommended in various agricultural soils to obtain a comprehensive understanding of the extent of MP pollution and to examine the combined effects of heavy metals and MPs on the soil fauna.

## Electronic supplementary material

Below is the link to the electronic supplementary material.

Supplementary Material 1

## Data Availability

All data generated or analyzed during this study are included in this published article and its supplementary information file (S1).
